# The role of social support and spiritual wellbeing in predicting suicidal ideation among marginalized adolescents in Malaysia

**DOI:** 10.1186/s12889-019-6861-7

**Published:** 2019-06-13

**Authors:** Norhayati Ibrahim, Normah Che Din, Mahadir Ahmad, Noh Amit, Shazli Ezzat Ghazali, Suzaily Wahab, Nor Ba’yah Abdul Kadir, Fatimah Wati Halim, Mohd Radzi Tarmizi A. Halim

**Affiliations:** 1 Health Psychology Programme, Faculty of Health Sciences, Universiti Kebangsaan Malaysia, Kuala Lumpur, Malaysia; 2 Psychiatry Department, Faculty of Medicine, Universiti Kebangsaan Malaysia Medical Centre, Bandar Tun Razak Cheras, Malaysia; 3 Centre of Human and Societal Well-being, Faculty of Social Sciences and Humanities, Universiti Kebangsaan Malaysia, Bangi, Selangor Malaysia

**Keywords:** Suicidal ideation, Adolescents, Spiritual wellbeing, Social support, Family support

## Abstract

**Background:**

The high number of adolescents and young adults harbouring suicidal ideation, as reported by the Ministry of Health Malaysia, is alarming. This cross-sectional study aims to examine the association between social support and spiritual wellbeing in predicting suicidal ideation among Malaysian adolescents.

**Methods:**

A total of 176 adolescents in selected urban areas in the states of Wilayah Persekutuan and Selangor were selected. The Suicide Ideation Scale (SIS) was used to measure the level of severity or tendency of suicidal ideation. The Multidimensional Scale of Perceived Social Support (MSPSS) was used to measure the perceived social support received by the respondent while the Spiritual Wellbeing Scale (SWBS) was used to measure the religious wellbeing (RWB), the existential wellbeing (EWB) and the overall score of spiritual wellbeing (SWB).

**Results:**

The study found that both RWB and EWB showed significant negative correlation with suicidal ideation. Similarly, support from family and friends also showed a negative correlation with suicidal ideation. Further analysis using multiple regressions showed that RWB and SWB, and family support predict suicidal ideation in adolescents.

**Conclusion:**

Spiritual wellbeing in combination with family support plays a major role in predicting suicidal ideation. Therefore, intervention for encompassing spirituality and family support may contribute to a more positive outcome in suicidal adolescents.

## Background

Suicide is the second leading cause of death among adolescents and young adults worldwide [1]. Although Malaysia was reported as one of the countries with the lowest completed suicide rate (0.6 per 100,000 persons) [2], the prevalence of suicidal behaviour among adolescents is alarming. In fact, research note that about 7% of adolescents in Malaysia have the intention to commit suicide and half of them have already attempted it [3]. A study by Maniam et al., 2013 [4] found that the overall prevalence of suicide ideation among Malaysians was 6.3%.

Suicide is an act that comprises three components: suicidal ideation, suicidal behaviour and completed suicide. However, a higher number of cases has been found for suicidal ideation compared to complete suicide. This statement is supported by a survey carried out by the Malaysian Ministry of Health, which found that 6.3% of people aged 16–19 and 20–24 have the highest suicidal ideation [5]. The reported numbers for suicidal ideation could be higher since there are unreported cases due to the stigma towards suicide and people with mental illness in developing Asian countries [6]. Furthermore, the shared sentiment of shame further reinforces the culture of stigmatization against suicide [7].

Currently, no specific theory has clearly and simultaneously discussed both spiritual/religion and social support as protective factors against suicidal behaviour. Most of the theories implicitly explain suicidal behaviour in term of spiritual/religion and social support, respectively. Theorist such as Durkheim discussed the theory of suicide in 1897 [8] but later in 1912 [9], he published another article that discussed the most primitive religious theory of human life. It appears to us that Durkheim had not clearly described the correlation on religion and suicide behaviour. Other theorist like Lakey and Cohen [10] also described the role of social support on physical and mental health but they did not clearly state that social support can act as protective factor for suicidal behaviour. Therefore, there is a need for a study that can examine a theoretical framework involving both spiritual/religion and social support in relation to suicidal behaviour. Osman and Gutierrez [11] suggested that risk factors and protective factors should be studied simultaneously. Despite the aforementioned limitations, some recent studies have made an attempt to provide the evidence of connection between spirituality, social support and suicidal behaviour to support their theoretical frameworks. Recent studies [12–14] have found that poor family support is associated with suicidal ideation and suicide attempts among adolescents. In fact, suicidal adolescents tend to perceive that their families are less engaged, less affectionate, and less confiding compared to either non-clinical adolescents or non-suicidal, depressed adolescents [15]. This condition occurs when there is a lack of mutual understanding between the adolescent and the parents, which also has a negative impact on how their children perceive the support they receive from their friends and their parents.

Gal et al. [16] studied the prevalence rates of suicidal behaviours among Arabs in Israel. They found that the prevalent rate of suicidal behaviours among Arabs was lower than Jewish-Israelis. Further analysis revealed that no differences were found among women aged 15–44 years old in relation to suicide. They concluded that although the rates of suicide behaviours were relatively high, the completed suicide behaviours were lower among Arabs as compared to Jews. It seems that their religion protects them from committing suicide. Meanwhile, another factor that is very important, and, historically, has received little attention in the suicide literature is spirituality. According to Moreira-Almeida et al. [17], research on the relationship between spirituality and religiosity, and suicidal behaviour is relatively neglected. Even the results on the relationship between spirituality and mental illness have been found to be less consistent. Some studies showed decreased odds of mental illness [18–22] while others showed either increased mental illness [23, 24] or no association between these two variables [25]. However, in general, studies have shown that people who report being more religious report lower levels of suicide ideation, and that people who report being less religious report greater suicide ideation [26–29].

Nevertheless, there are limited number of studies examining the role of social support and spirituality in predicting suicide ideation in developing countries. Several studies have investigated factors associated with suicidal ideation among adolescents in Malaysia. For example, Ibrahim et al. [30] studied the psychological factors as predictors of suicidal ideation among adolescents in Malaysia. The results showed that there were significant correlations between depression, anxiety and stress, and suicidal ideation. Khan et al. [31] investigated the influence of psychological factors on suicide ideation among Malaysian and Indian adolescents. The results showed significant differences between Malaysian and Indian adolescents in terms of hopelessness, negative effects, suicide ideation, depression and academic stress. Mustaffa et al. [32] investigated depression and suicidal ideation among university students and found a significant correlation between the two factors. This is supported by findings from Ahmad Nabil & Suarn [33]. At present, there are limited studies to assess the relationship between social support and spirituality, and suicide ideation issues among the Malaysian population. In realizing that there is a need to obtain a better understanding about the association of these two factors on suicidal ideation among local adolescents, this study aims to find out the correlation and to determine the influences of these two protective factors - social support and spiritual wellbeing on suicide ideation among marginalized adolescents in Malaysia. Although research has examined adolescents in general population, there is limited knowledge on marginalized adolescents. Understanding of marginalized adolescents is important as they may have limited access to health care including mental health services. This could inform the policy in promoting early screening and prevention of suicide risk among marginalized adolescents in Malaysia [34].

## Methods

### Research design

A cross-sectional study was conducted in selected urban areas in the states of Federal Territory of Kuala Lumpur and Selangor, Malaysia. The participants were selected based on simple random sampling method (where lists of house numbers were pooled and 200 house numbers were withdrawn randomly). The sample size was calculated based on Tabachnick and Fidell [35] formula.

### Participants

This study involved 176 adolescents aged between 13 and 19 years and focused on adolescents who lived in the city with a low-income family earning less than RM3000 per month. Informed consent and assents were obtained from individual participants in the study.

### Measurement instruments

The Suicide Ideation Scale (SIS) was constructed by Rudd in 1989 [36], and contains 10 items that measure the level of severity or tendency of suicide ideation among adolescents and young adults. Each item is a statement about suicidal behaviour ranging from suicide ideation to suicide attempts. Each of these statements is measured using a five-point Likert scale starting from 1 (never) to 5 (very often) depending on how often the respondents feel or behave according to the statement in the last year. Conceptually, this scale is capable of measuring the suicidal behaviour beginning with suicide ideation (covert suicidal thought) until suicide attempt, and, finally, the actual suicide. In terms of reliability, the internal consistency of SIS is is high with alpha Cronbach of 0.90 and the correlation between items ranged from 0.49 to 0.78 [34]. Meanwhile, for the construct validity, the SIS is correlated with the Center for Epidemiologic Studies Depression Scale ([CES-Dl], Radloff, [37] to produce r = 0.55, and the scale of hopelessness (Beck Hopelessness Scale; Beck, Weissman, Lester, & Trexler, 1974) [38] with r = 0.49. The score for this test instrument varies from 10 (no suicide ideation in the last year) up to 36 (serious suicide ideation benchmark) with a mean of 12.04 and standard deviation 3.73. Serious suicide ideation is defined by the score for the SIS being more than the mean and the standard deviation. The reliability of SIS in the present sample is 0.94.

### Multidimensional scale of perceived social support (MSPSS)

The MSPSS was developed by Zimet, Powell, Farley, Werkman & Berkoff in 1990 [39] to measure the perceived social support received by respondents. Social support perception was assessed through three dimensions: i) the support of the family, ii) the support from friends, and iii) the support from a special one. The internal consistency for each dimension and the overall testing instrument shows a very good reliability (alpha Cronbach) of 0.91 for the support from a special one, 0.87 for family support and 0.85 for support from friends, while the overall reliability stands at r = 0.88. Accordingly, the results of the re-test produce reliabilities of 0.72 for the support from a special one, 0.85 for family support and 0.75 for support from friends, as well as 0.85 for the overall. The Malay version of MSPSS also showed good internal consistency (Cronbach α of 0.89) and validity. The reliability for each subdomains is high for perceived family support (0.88), friends’ support (0.82) and significant others (0.94). The scores of the total subscales for MSPSS-M were negatively correlated with the depression subscale in DASS (*p* < 0.05) [40].

### Spiritual wellbeing scale (SWBS)

The SWBS was developed by Paloutzian and Ellison in 1982 [41], and comprised of 20 items that measure the two subscales of religious wellbeing (RWB) and the existential wellbeing (EWB), as well as an overall score for spiritual wellbeing (SWB). SWB measures the overall religious wellbeing. RWB also measures how the respondents view their relationship with God and the degree of satisfaction they gain from a positive relationship with God. On the other hand, EWB measures the satisfaction with life and the purpose of life. Each subscale (RWB and EWB) contains 10 items and is combined to produce the SWB (20 items). This test instrument was translated and validated by Imam et al. [42] in which the word ‘God’ from the original items was replaced with the word ‘Allah’. However, for this study, the researcher used the term ‘Tuhan’ since the study samples involved respondents from different religions in Malaysia. Each item is measured using a 6-point Likert scale starting with ‘strongly disagree’ (1) to ‘strongly agree’ (6). The original reliability values ranged between r = 0.73 and 0.99, while the internal consistency is between r = 0.78 and 0.94 [43]. For the Bahasa Malaysia edition, the internal consistency for SWB is at r = 0.88, RWB at r = 0.86 and for EWB at r = 0.81. Apart from that, the convergent validity found that SWB is significantly correlated with RWB (r = 0.89) and with EWB (r = 0.90).

### Data analysis

The raw data were keyed into the Statistical Package for the Social Sciences (SPSS) version 21. Descriptive analysis was used to describe the demographic data and distribution. Pearson’s correlation was carried out to measure the relationship between the social support and spiritual wellbeing with suicide ideation. Stepwise multiple regression was conducted to predict the influence of social support and spiritual wellbeing on the suicide ideation of adolescents.

## Results

### Demography

A total of 176 adolescents who lived in the city with a family income below RM3000 and aged from 12 to 19 were involved in this study. Table 1 showed that there were 105 (60%) males and 71(40%) females. The majority of the respondents were Malay (85%) and Muslim (88%). Most of them were still schooling (88%). The rate of suicide ideation using one screening item showed that 6.8% of the respondents had suicidal ideation.

**Table 1:** Socio-demographics of respondents

Demographics	n	Percentage
Gender		
Male	105	59.7%
Female	71	40.3%
Age		
12–14	40	22.7%
15–16	55	31.3%
17–19	81	46.0%
Ethnicity		
Malay	149	84.7%
Non-Malay	27	15.3%
Religion		
Muslim	155	88.1%
Non-Muslim	21	11.9%
Education Level		
Diploma and cert	20	11.4%
SPM	56	32.0%
PMR	59	33.7%
UPSR	35	20.0%
Non schooling	5	2.9%
Suicide ideation screening using one item		
Yes	12	6.8%
No	164	93.2%

A total of 68.7% respondents did not have suicide ideation, while the other 31.3% adolescent respondents harboured suicide ideation or had attempted suicide within the last year (Table 2). Religious wellbeing (RWB) and existential wellbeing (EWB) were significantly negatively correlated with suicide ideation (Table 3). In other words, higher religious wellbeing and existential wellbeing were related to less suicide ideation in these adolescents. The total score for spiritual wellbeing also showed a negative correlation with suicidal ideation. The results also indicated that there were negative correlations between the support that the adolescents received from families and friends with suicide ideation. However, there was no significant correlation between support from a special one and suicide ideation.

**Table 2:** Prevalent rates of suicide ideation using SIS

	Frequency	Percentage
No suicide ideation	121	68.7
Have suicide ideation up to suicide attempt	55	31.3
Total	176	100

**Table 3:** Correlation between spiritual wellbeing, social support, and suicide ideation

	Mean	SD	r
Suicide ideation	15.08	7.91	
Religious wellbeing	49.21	11.06	−0.30**
Existential wellbeing	33.56	9.35	−0.19*
Total score for Spiritual wellbeing	82.76	14.77	−0.34**
Support from a special one	20.27	6.57	−0.06
Support from family	20.29	6.18	−0.21**
Support from friends	20.47	5.08	−0.21**
Total support	61.02	14.17	−0.18*

*p < 0.05; **p < 0.001

Finally, multiple regression using the stepwise method was performed to determine the influence of religious wellbeing and existential wellbeing, and family support and friend support on suicide ideation. The results showed that religious wellbeing was a good predictor and this factor contributed 8.7% of the variance in suicide ideation. Existential wellbeing was also one of the significant predictors of suicide ideation. However, this variable only contributed 3.2% of the variance in suicide ideation. Both family support and friend support were negatively correlated with suicide ideation but only family support was a predictor of reducing suicide ideation among adolescents and contributed 2.2% of the variance (Table 4).

**Table 4:** Predictors of suicide ideation

Model	Standardized Coefficient Beta	t	sig	R square
Step 1		9.74	0.000	
Constant				
Religious wellbeing	−0.30	−4.07	0.000	0.087
Step 2				
Constant	12.54	9.74	0.000	
Religious wellbeing	−0.29	−4.03	0.000	0.119
Existential wellbeing	−0.18	−2.52	0.012	
Step 3				
Constant		9.61	0.000	
Religious wellbeing	−0.27	−3.71	0.000	0.141
Existential wellbeing	−0.16	−2.31	0.022	
Family support	−0.15	−2.07	0.040	

## Discussion

Although results from the single item scale showed that 6.8% of the adolescents rated themselves as having suicide ideation, more comprehensive questionnaire showed that 31.3% of the adolescents rated themselves as having suicide ideation. The high prevalence of reported suicide ideation could be attributed to the sensitivity and high variation of item using standardized questionnaire of the Suicide Ideation Scale (SIS) compare to a single item. Single item measure of suicide ideation may contribute to misclassification and measurement problem such as the likelihood of statistical decision error [44].

The considerably high prevalence of reported suicidal ideation among adolescents in the present sample also could be explained by their social environment. The majority of adolescents in the present sample are from marginalized population, dwelling in a high risk environment of urban city and with low-income families earning less than RM3000 per month. People who live in Malaysia urban areas with a cumulative family wage of less than RM3000, are classified as poor urban population [45]. Socio-economic status is one risk factors for suicide ideation and attempt. Findings showed low socio-economic status could be associated with suicide ideation and suicide attempts [46]. Study by Qin, Agerbo, and Mortensen [47] also found that low socio-economic status is an important risk factor for suicide, especially in males. A recent study by Page et al. [48] found that a lower socio-economic position during childhood is associated with the subsequent risk of self-harm and suicidal intent in adolescence.

In terms of relationship, the present study found that both religious wellbeing (RWB) and existential wellbeing (EWB) was negatively correlated with suicide ideation. Support from family and friends was also negatively correlated with suicide ideation. Besides that, the regressions analysis showed that religious wellbeing (RWB) and spiritual wellbeing (SWB), and family support predicted suicide ideation in adolescents. The result suggests that family support acts as a protective factor of suicidal ideation. This is because adolescents with good family support believe they are loved, valued, and family is a part of a social network they access in times of need [49]. This is in line with attachment theory where the parents are responsive to the adolescent’s needs and emotional availability [49, 50]. These experiences, in return, could produce a sense of being socially connected and feeling supported, thus lower the risk of suicidal ideation. A few study studies have shown that the perceived quality of family relationship is an important risk or protective factor of suicide in clinical and community samples of adolescent [51, 52]. This has been indicated by theory of social support where the health effects of social support cannot be separated from relationship processes between parents and adolescents in times of crisis such as in dealing with personal problem including suicide ideation. These findings are consistent with Consoli et al. [53] which they found that the family factor, especially perceived negative relationship with the parents is associated with suicidal behaviour. Hong and Jeon [54] also reported that the causes of suicidal ideation and suicidal attempts among adolescents are negative relationship and interaction with parents. At this age, adolescents need a good relationship, especially with family, to enhance their self-esteem, and enable them to express and share their feelings and problems. Such support is not only a protective factor, but can also enhance their general wellbeing [55]. The findings of the present study are also in line with Kerr et al. [12] and Amit et al. [56]. Their findings showed that family support is negatively related to hopelessness, depressive symptoms, and suicidal ideation among the respondents. Durkheim also emphasized on the importance of family support using the concept of social integration, varying family circumstances as well as the role of religious integration to understand suicide. Durkheim theorized that insufficient social integration enhances individualism which encourage egoistic suicide, while a society that is unable to regulate individuals’ naturally unlimited ambitions would aspire anomic suicide [8]. Durkheim used control theory to explain suicide by assuming that delinquent acts results when an individual’s bond to society including the family is weak or broken.

In addition, the findings suggest that spiritual wellbeing is a protective factor of suicide ideation. In the present study, 85% of the respondents are Muslims. As a Muslim, suicide ideation is prohibited. Suicide in Islam is considered as a very grave sin. Relating the prevalence rates of suicide with religious beliefs, previous studies showed that the prevalence rates of suicide among Muslims are low [16]. Muslims believe that through the relationship with God and engagement in congregational prayers in mosques, people may seek help from religious teacher or leader in addressing their problems. However, some adolescents neglect spiritual support due to their busy modern lifestyle and working parents while others blame God for all the problems that occur. According to Taliaferro et al. [57], who explored whether specific dimensions of spiritual wellbeing (religious wellbeing and existential wellbeing) relate to reduced suicide ideation, existential wellbeing is an important factor associated with lower levels of suicide ideation among college students. A recent study by Shaheen et al. [58] also found that there is a significant negative correlation between overall spirituality scores and suicide ideation. As religion may offer a worldview about life and self to believers, youth who are highly religious are more optimistic and believe that they can overcome obstacles, and, therefore, are less prone to suicidal behaviour. Smith et al. [59] explained further on the role of spiritual well-being and family support among youth. They found that family consonance (spiritual activity) and family church network significantly predict adolescents spiritual well-being and resilience. Thus, action should be taken by the community, parents and friends alike to encourage themselves and the young people surrounding them to be more involved in spiritual based activities to divert their mind from negative thoughts and live a healthy life.

The findings provide some insight that both spiritual/religion and social support can contribute a lot in saving lives. Considering the findings from this study with the supports from previous studies, spiritual wellbeing and family support should be embedded and emphasised in the existing psychotherapy approaches and pharmacological treatments for suicidal adolescents and young adults. The application of spiritual believe, religious activities, good and communicable social support from the loved ones makes the suicidal person feel more belongingness, faithful, accept the truth and feel less burdensome and perhaps, and feeling connected to God and the loved ones are important in working with patients with suicide risks as had been suggested by McLean et al. [60]. The findings of this study path a more directed strategy to work with adolescents and young adults in preventing and intervening suicide. The strategies that emphasise on spiritual/religious wellbeing and family support can be implemented at community/societal level through educational system, religious activities and youth development activities. On the intervention basis, more training and skills development for suicide intervention should be directed towards enhancing family support system and existential issues among adolescents and young adults. Therapists and individuals who are dealing with this issue should be well-trained in the related areas.

This study has some limitations. With regard to research design, causal relationship is unable to be established whereby all measures were taken at one point in time means it was impossible to determine the time order of the variables. Therefore, it was not known whether lack of social support and religious wellbeing both preceded the development of suicidality. Our findings are limited to the Malay Muslim adolescents of low socioeconomic status in the community. Therefore, the results may not be generalized to include other adolescents of clinical populations. This study did not examine the interaction between social support and religious well-being to test mediation or moderation of the variables.

For future research, it will be beneficial to include multiple risk factors and protective factors to assess aetiology of suicide ideation among young people in Malaysia. It is also important to refine previous theoretical frameworks in relation to the contribution of social and psychological factors to suicide ideation in the context of Malaysia and thus providing a knowledge on  specific cultural context of suicide ideation in Malaysia.

## Conclusion

The findings corroborate literature on role of religious wellbeing, existential wellbeing and support from family and friends as protective factors for suicide ideation among adolescents. It also extends knowledge on predicting role of religious wellbeing, spiritual wellbeing and family support in predicting suicide ideation among adolescents in Malaysia. Future research may focus on role of religious wellbeing and spiritual wellbeing in predicting suicide ideation among other ethnic groups and of other religious affiliations in Malaysia.

## Abbreviations

ALSPAC: Avon longitudinal study of parents and children
CES-Dl: Centre for epidemiologic studies depression scale
EWB: Existential wellbeing
MSPSS: Multidimensional Scale of Perceived Social Support
RWB: Religious wellbeing
SIS: Suicide Ideation Scale
SPSS: Statistical Package for the Social Sciences
SWB: Spiritual wellbeing
SWBS: Spiritual Wellbeing Scale
UKM: National University of Malaysia
